# Draft genome sequence of *Talaromyces trachyspermus*, a biocontrol fungus isolated from broomrape

**DOI:** 10.1093/g3journal/jkaf280

**Published:** 2025-12-29

**Authors:** Roghayeh Hemmati, Aria Dolatabadian, Sobhan Saeedi, Jacqueline Batley

**Affiliations:** Department of Plant Protection, Faculty of Agriculture, University of Zanjan, Zanjan 45371-38791, Iran; School of Biological Sciences, The University of Western Australia, Crawley, WA 6009, Australia; Department of Plant Protection, Faculty of Agriculture, University of Zanjan, Zanjan 45371-38791, Iran; School of Biological Sciences, The University of Western Australia, Crawley, WA 6009, Australia

**Keywords:** antifungal activity, biosynthetic gene clusters, cell wall-degrading enzymes, Illumina, Penicillium, whole-genome sequencing, genome assembly

## Abstract

Applying antimicrobial compounds derived from microorganisms for plant disease management is one of the objectives of sustainable agriculture. The genus *Talaromyces* is known for its species' ability to produce a diverse group of antimicrobial compounds. For example, *T. trachyspermus* has been reported to produce secondary metabolites, “cell wall degrading enzymes,” and plant growth-promoting factors. Identification of novel promising metabolites and enzymes from *T. trachyspermus* is still in its infancy. Also, there is a lack of information about the genomic resources for its secondary metabolites and hydrolytic enzymes. Therefore, this study aimed to analyze the genome of a biocontrol isolate of this species to investigate its biocontrol mechanisms at the genomic level, focusing on secondary metabolites and “cell wall degrading enzymes.” The whole genome of *T. trachyspermus* isolate IRAN 3054C, obtained from necrotic *Orobanch ramosa* stems in Iran with biocontrol ability, was sequenced using the Illumina platform. We performed both *de novo* and resequencing analyses of the genome, obtaining a 31.3 Mb assembly. The abundance of protein groups associated with biocontrol activities was assessed in the studied genome. Fungismash was used to detect and annotate secondary metabolites. The analysis revealed the presence of several secondary metabolite biosynthesis gene clusters, with a high frequency of polyketide synthases (T1PKSs) and nonribosomal peptide synthetases, which are known to produce bioactive compounds with antimicrobial properties. Among the identified putative secondary metabolites, fusarin, YWA1, dimethylcoprogen, and squalestatin S1 exhibited the highest similarity to known compounds. Furthermore, sequences similar to phyllostictine A/B indicate putative potential herbicidal properties. The genome also had domains for enzymes involved in phosphate solubilization, siderophore production, and fungal cell wall degradation, which are essential for biocontrol and plant growth promotion. Our findings highlight the genomic richness of *T. trachyspermus* IRAN 3054C for biocontrol. Further metabolomics studies are needed to validate the actual production of these secondary metabolites and explore their functional roles in biocontrol.

## Introduction

*Talaromyces*, belonging to Ascomycota, Eurotiomycetes, and Trichocomaceae, is a teleomorphic form for some *Penicillium* species. Based on multi-locus phylogenetic analysis, the genus is classified into 7 sections (Yilmaz et al. 2014). *T. trachyspermus* (Shear) Stolk and Samson 1972, is a species within the *Talaromyces* section *Trachyspermi*, distinguished by slow-growing, floccose white to yellow mycelium, mono-verticillate conidiophores, smooth ellipsoidal conidia, and abundant globose to sub-globose cleistothecial ascomata with ellipsoidal ascospores (Stolk and Samson 1972). *Talaromyces* species are distributed in nature and different environments. They have been isolated from various substrates such as soil, food, and plant materials. Some species of this genus produce anticancer, antibacterial, and antifungal compounds; others are important producers of lignocellulolytic enzymes and natural pigments (Fu et al. 2024). Some plant-associated species have been reported as endophytes (Yilmaz et al. 2016; Quan et al. 2024), whereas some are plant-pathogenic fungi (Sun et al. 2020). Some species are mycotoxin producers, and others cause food spoilage (Yilmaz et al. 2014).

Several reports show the antagonistic effects of Talaromyces species against plant pathogenic fungi and/or their plant growth-promoting effects (Fahima and Henis 1995; Nagtzaam 1998; Manoch and Dethoup 2011; Abdel-Rahim and Abo-Elyousr 2018; Sahu et al. 2019; Goh et al. 2020; Zhao et al. 2022; Farhat et al. 2023; Fu et al. 2024; Zhang et al. 2024). Among those species, *T. trachyspermus* is an important *Talaromyces* species of antagonistic fungi. There are reports of its biocontrol effects against plant pathogenic fungi, including *Alternaria brassicicola*, *Colletotrichum capsici*, *Pythium aphanidermatum*, *Rhizoctonia solani*, *Sclerotium rolfsii* and *Sclerotinia sclerotiorum*, *Alternaria alternata, Alternaria arborescens, Botryosphaeria dothidea*, and *Colletotrichum gloeosporioides* (Dethoup et al. 2015; Sahu et al. 2019; Zhao et al. 2020). One isolate of *T. trachyspermus*, isolated from the necrotic tissue of broomrape stems (*Orobanche* spp.) in Iran, caused a significant reduction in the number of tubercles, indicating its potential as a biocontrol fungus against this parasitic plant (Hemmati and Gholizadeh 2019). Several other fungal species, specifically within the *Fusarium* genus, have been reported as mycoherbicidal fungi against *O. ramosa* (Abouzeid et al. 2004; Boari and Vurro 2004; Ghannam et al. 2007; Abouzeid and El-Tarabily 2010). *T. trachyspermus* isolated from the medicinal plant *Withania somnifera* (winter cherry) showed high production of hydrolytic enzymes, protease, chitinase, amylase, cellulase, and pectinase, which are required for biocontrol activities. In the meantime, this isolate produced high levels of indole acetic acid, siderophore synthesis, and phosphate solubilization activities important for plant growth promotion (Sahu et al. 2019). This species has been reported as a successful producer of spiculisporic acid (SA) as a fatty acid-type biosurfactant useful in the cosmetics industry (Moriwaki-Takano et al. 2021).

*T. trachyspermus* has gained attention recently due to its production of various bioactive secondary metabolites with antimicrobial properties (Zhai et al. 2016; Farhat et al. 2022). Secondary metabolites (SMs) are a group of low molecular weight natural products that are not vital for their producers but can increase their fitness in different habitats. Consequently, they can increase their survival in competitive environments (Conrado et al. 2022). Fungal secondary metabolites are divided into 4 classes: polyketides, non-ribosomal peptides, terpenoids, and “shikimic acid derived” compounds (Pusztahelyi et al. 2015). These secondary metabolites can act as antimicrobial agents against bacterial and fungal pathogens, including *Fusarium oxysporum* and *Phytophthora nicotiana* (Yao et al. 2023). The genes encoding enzymes for synthesis of secondary metabolites are grouped into gene clusters. The secondary metabolites of *Talaromyces* mainly include alkaloids, peptides, lactones, polyketides, and miscellaneous structure-type compounds (Zhai et al. 2016). Genomic analysis of some of the *Talaromyces* species has provided useful information about the genetic determinants of secondary metabolite biosynthesis. *Talaromyces pinophilus* strain 1 to 95 genome was sequenced and revealed a high number of secondary metabolite gene clusters. They discovered 68 gene clusters for secondary metabolism consisting of type I polyketide synthase genes and nonribosomal peptide synthetase (NRPS) genes. These gene clusters are important in producing a variety of bioactive compounds, including antimicrobials essential for antagonistic activity (Li et al. 2017). In another study, the endophytic fungus *Talaromyces* sp. strain DC2 genome had 20 biosynthetic gene clusters for secondary metabolite production. The gene list indicates that the strain can produce a broad spectrum of secondary metabolites (Quan et al. 2024). The genome of *T. albobiverticillius* Tp-2 contains 62 distinct gene clusters for secondary metabolite biosynthesis. This strain showed a high genomic capacity to generate different bioactive compounds, including pigments with the potential of industrial applications (Wang et al. 2023). The production of “cell wall degrading enzymes” (CWDEs), including proteases, chitinases and glucanases, is one of the essential mechanisms that fungal biocontrol agents employ against plant pathogenic fungi (Inglis and Kawchuk 2002). It has been demonstrated that mycoparasite species of *Trichoderma* have more proteases, a higher number of “chitinase encoding genes,” and more copies of glucanases compared with saprophytic species (Gruber and Seidl-Seiboth 2012).

Several works have reported high potential for production of “biomass degrading enzymes” by different Talaromyces species. Genomic analysis of *T. pinophilus* revealed that this species has 803 genes to encode enzymes acting on carbohydrates; among them, 39 enzymes were cellulose-degrading, and 24 were starch-degrading (Li et al. 2017). A “whole genome” analysis of *Talaromyces* sp. strain DC2 revealed that the genome of this strain has a total of 149, 227, 65, 153, 53, and 6 genes responsible for cellulose, hemicellulose, lignin, pectin, chitin, starch, and inulin degradation, respectively (Quan et al. 2024). There are several works on biochemical identification and product optimization of some important enzymes from different species of this genus, including xylanase production by *T. amestolkiae* on agro-industrial wastes (Barbieri et al. 2022), GH51 α-l-arabinofuranosidase production by *T. leycettanus* (Tu et al. 2019), industrially significant proteases, including thermostable aspartic protease from *T. leycettanus* (Guo et al. 2020), a novel AA14 LPMO from *T. rugulosus* with strong oxidative activity on cellulose, xylan, and xyloglucan (Chen et al. 2024), and identification of a gene encoding an extracellular β-galactosidase of *T. cellulolyticus* (Orleneva et al. 2022).

Genome-wide analysis and other bioassay and biochemical experiments, such as metabolomics, will help to understand the biocontrol mechanisms, such as antibiosis and parasitism, and the genomic basis underlying these mechanisms. For *T. trachyspermus*, only one genome assembly of 32Mb size belonging to strain 4014 isolated from a medicinal plant (Sahu et al. 2019). There is no genome annotation and published data analysis for this whole-genome sequence. Therefore, there is a lack of information regarding the genomic resources for biocontrol determinants such as secondary metabolites, “cell wall degrading enzymes,” and plant growth promotion factors for this species. In the current study, we performed “whole-genome” sequencing for a biocontrol isolate of this species, IRAN 3054C, which we have isolated from broomrape in Iran (Hemmati and Gholizadeh 2019). This isolate has shown antifungal effects, biocontrol activity against *O. ramosa,* and plant growth promotion phenotypes (Hemmati and Gholizadeh 2019, and unpublished data). As a part of whole-genome data analysis, we aimed to predict proteins and secondary metabolites, specifically carbohydrate-active enzymes (CAZYmes) and polyketides, which are associated with biocontrol. The genomic analysis of this fungal isolate will help us to have a deeper and more precise understanding of its capacities as a promising biocontrol agent.

## Materials and methods

### The origin of the isolate and preparation of fresh cultures

This study used a *T. trachyspermus* isolate (IRAN 3054C) obtained from broomrape plants showing stem rot symptoms. This isolate has previously been reported as a potential biocontrol agent against broomrape due to its effects on broomrape seedlings and a significant reduction in the number of emerging shoots of this parasitic plant in tomato pots under greenhouse conditions (Hemmati and Gholizadeh 2019). The dehydrated form of this isolate was preserved at −20 °C as dried pellets of potato dextrose agar (PDA) cultures of the fungus. A small pellet was placed on a Petri dish containing PDA and incubated in darkness at room temperature (24 ± 2 °C). After 10 d, the fresh cultures were ready for DNA extraction.

### DNA extraction, qualification, and quantification

Seven-day-old colonies were stored at −70 °C for 24 h before DNA extraction. Then, the frozen mycelia were harvested from the culture media by scraping the hyphal layer from the surface of the agar medium and ground by using sterilized porcelain mortar and pestel. The DNA was extracted using the CTAB method (Möller et al. 1992). The total genomic DNA was assessed for quality using 1% agarose gel electrophoresis and was quantified using a Qubit 3.0 Fluorometer (Life Technologies, USA).

### Library preparation and whole-genome sequencing

The minimum volume of 10 µl of each DNA sample, with a minimum concentration of 10 ng. µl^−1^, was sent to AGRF (Australian Genome Research Facility) for library preparation and “whole-genome” sequencing. Sequencing was performed on the Illumina platform with 2 paired-end reads (150 bp). The initially requested sequencing coverage was 20×, but the sequencing run generated ∼1.59 Gb of data for a ∼31.3 Mb genome, corresponding to ∼51× theoretical coverage. After mapping to the reference genome (strain 4014), the effective average coverage was ∼46×, with 95.87% of reads successfully aligned.

### Whole-genome data analysis

#### Resequencing analysis

A FASTA format of a reference genome for *T. trachyspermus* (BUMICRO_TalaroTrachy_1.1, strain 4014) was downloaded from NCBI. The related FASTA file was uploaded to “use.Galaxy.org”; an annotated GFF3 file was created using Augustus. Augustus was run with the built-in training set using the pre-trained *Aspergillus nidulans* model. Gene models were predicted as complete, independent on both strands, and without the use of extrinsic hints. Softmasking was enabled, and transcripts containing in-frame stop codons were excluded. Predictions were limited to coding sequences only, without inclusion of untranslated regions. No additional species-specific training was performed.

The resulting file was imported into the CLC Genomics Workbench 20.0 (QIAGEN 2020). For mapping to the reference genome, the genome of our biocontrol isolate was aligned with the reference genome of *T. trachyspermus* (strain 4014) using CLC Genomics Workbench. Variant calling was performed to identify single-nucleotide polymorphisms and small insertions/deletions (indels) between the biocontrol isolate and the reference genome.

#### *De novo* assembly

The raw Illumina paired-end reads (150 bp) underwent quality control and preprocessing steps in the CLC Genomics Workbench (version 20, Qiagen). This involved assessing sequencing quality and trimming low-quality bases. A *de novo* assembly was performed on the resulting reads in the CLC Genomics Workbench with the following parameters: automatic word size and bubble size detection, minimum contig length of 200 bp, and scaffolding enabled with automatic detection of paired-end distances. Default settings were used for all other parameters. The resulting assembly was further evaluated based on N50, genome length, GC content, and BUSCO completeness scores. This Whole Genome Shotgun project has been deposited at DDBJ/ENA/GenBank under the accession JBNAEF000000000. The version described in this paper is version JBNAEF010000000. The associated BioProject and BioSample accessions are PRJNA1243591 and SAMN47626500, respectively. The percentage of repetitive sequences in the genome was identified using the Red repeat masking pipeline (Red, version 2018.09.10, Girgis 2015) available on the Galaxy web-based analysis platform. As a gene prediction tool on the Galaxy platform, Augustus was used to predict protein-coding genes in both the studied draft genome and the reference genome.

Further quality assessments were performed by using the BUSCO v5.5.0 pipeline in the Galaxy web-based analysis platform with “eurotiales_odb10” and used as reference datasets (https://busco.ezlab.org/). The BUSCO pipeline was also used to predict and annotate protein-coding genes using the default e-value threshold of <1e−03. The BUSCO annotation pipeline utilized metaeuk (v5.34c21f2) to annotate the fungal assemblies (Levy Karin et al. 2020).

EggNOG (v2.1.8) in the online GALAXY platform was used to identify and annotate orthologous groups (OGs) using the default e-value threshold of 10^−3^. This analysis provided several forms of functional annotation, including Gene Ontology terms, KEGG pathways, SMART/Pfam domains, and clusters of orthologous groups of proteins (COGs) (Tatusov et al. 2000). To analyze the Gene Ontology (GO) terms for all the predicted Pfam domains, we mapped them against the data in STRING (STRING: functional protein association networks [string-db.org]). The web-based program Categorizer was used to classify the GO terms for all the identified domains (Hu et al. 2008). Genes belonging to 13 protein groups were selected based on their reported involvement in biocontrol-associated functions, such as parasitic activities, and their abundance across the genome was recorded for our studied isolate.

Fungismash (https://fungismash.secondarymetabolites.org/) was used to detect and functionally annotate secondary metabolites encoding gene clusters (SM-BGCs) using the strict detection strictness setting (Blin et al. 2021).

## Results

### The results of variant calling for re-sequencing analysis

After mapping the reads to the *T. trachyspermus* (strain 4014) as the reference genome, the total number of variants was 312,928, including 96,634 insertions, 11,121 deletions, 190,295 single-nucleotide variants (SNVs), 12,671 multinucleotide variants (MNVs), and 2,207 replacements. Among the variants, 28,571 (9.13%) were associated with amino acid changes in the studied isolate, including 132 replacements, 10,893 insertions, 15,666 SNV, 1,243 MNV, and 639 deletions.

### Genome features following *de novo* assembly

A 31.31 Mb genome was generated with 20-fold coverage. The size was smaller than the estimated genome size of 32 Mb for *T. trachyspermus* strain 4014, which serves as the reference genome for this species. Five hundred fifty-two contigs covered the genome. The N50 and N75 sizes of the contigs were, respectively, 385.98Kb and 176.13Kb. The GC content was 47.3%. The BUSCO completeness score for the studied genome was 92.26%, with a total of 4,191 BUSCO genes identified (Table 1).

**Table 1.** Summary of *Talaromyces trachyspermus* strain IRAN 3054C draft genome sequencing and assembly results compared to the known and annotated features of *T. trachyspermus* strain 4014 as the reference genome.

	Strain IRAN 3054C	Strain 4014 (reference genome)
Total sequenced bases	1,594,985,700	Not known
Mean read length	150	Not known
Number of reads	10,633,238	Not known
Contigs	552	14
Largest contig (bp)	1,321,046	Not known
Total length	31,309,464 (bp)	32,000,000 (bp)
GC (%)	47.3	47
N50	385,981 (bp)	3.8 Mb
N75	176,132	Not known
Protein-coding genes (from Augustus)	10,106	7905
Repeats content (%)	59	Not calculated
Busco annotation results (%)		
Complete	92.26	59.1
Complete and single copy	91.26	58.9
Complete and duplicate	1	0.1
Fragmented	0.85	18.3
Missing	6.89	22.6

### Clusters of orthologous groups

**Fig. 1.**
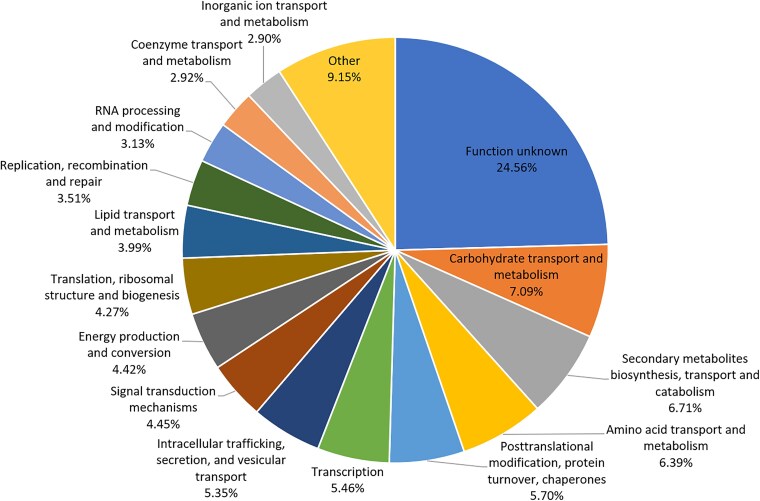
Clusters of orthologous groups (COG) functional categories and their abundances (%) in the *Talaromyces trachyspermus* Iran IRAN 3054C genome. For display limitations, 9 of the COG functional categories with a mean relative abundance of less than 2% were grouped into the category of “Other.”

Functional annotation analysis (EggNOG) detected 8,503 OGs in the *Talaromyces* strain; among them, 95.2% of OGs were assigned a COG annotation (COGs of proteins). As depicted in Fig. 1, the COG functional categories with the highest mean relative abundances included “function unknown” (24.56%), “carbohydrate transport and metabolism” (7.09%), “secondary metabolite biosynthesis, transport, and catabolism” (6.71%), and “amino acid transport and metabolism” (6.39%).

**Fig. 2.**
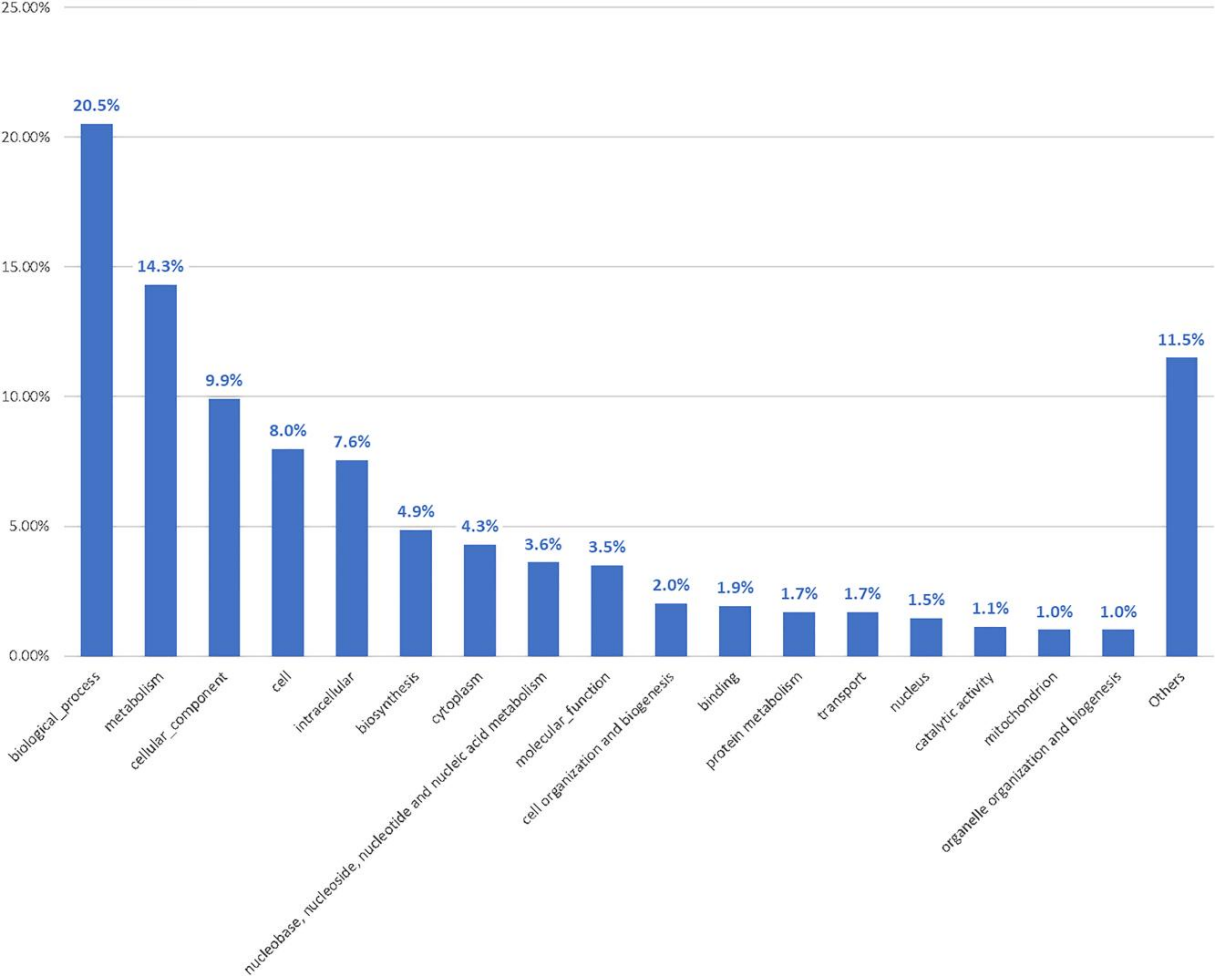
Go terms classification count resulted by mapping identified GO terms to GO_slime ancestor terms, available at CateGOrizer.

By mapping identified GO terms for the studied isolate to 127 of the GO_slime ancestor terms by a single count, available in CateGOrizer (Hu et al. 2008), 182 GO terms were classified into biological processes, 127 into metabolism, 88 into cellular components, and 31 into molecular functions. Overall, 301 unique terms were assigned to at least one of the 45 GO_slime classes (Fig. 2).

Among the Pfam domains, we identified several domains for producing carbohydrate-active enzymes (CAZymes) and fungal “cell wall degrading enzymes” (FCWDEs). Peptidases had the most domains in our studied genome (count: 93). The second- and third- most frequent domains with fungal cell wall degrading activity or parasitism belonged to chitinases (count: 31), proteases (30), and lipases (30). Glucanases, another group of FCWDEs, had 13-count domains in the genome (Table 2).

**Table 2.** Pfam domains associated with carbohydrate-active enzymes, fungal “cell wall degrading enzymes,” and plant growth-promoting factors in the *Talaromyces trachyspermus* genome extracted from EggNOG output.

Pfam domain	Function/Process	The count number of domain
β-glucosidases	Synthesis and degradation of complex carbohydrates (CAZyme)	7
Glucanases	CAZyme^a^/FCWDEs^b^	13
Galactosidases	CAZyme	4
Chitinases (GH18)	CAZyme/FCWDEs	31
Chitosanases	CAZyme/FCWDEs	0
Mannosidases	CAZyme	10
α-L-Arabinofuranosidase	CAZyme	1
β-L-Arabinofuranosidase	CAZyme	3
Glycoside hydrolase: Xylanase	CAZyme	8
N-acetyl-β-D-glucosaminidase	CAZyme	1
Cellulolytic enzymes	CAZyme	3
Pectate lyase	CAZyme	6
Peptidase	Parasitism/FCWDEs	93
Protease	Parasitism/FCWDEs	30
Lipase	Parasitism	30
Siderophore synthase	PGP^c^	5
Indole acetic acid	PGP	0
Acid phosphatases	PGP	15
Alkaline phosphatases	PGP	2
Phytase	PGP	1

^a^Carbohydrate active enzymes.

^b^Fungal cell wall degrading enzymes.

^c^Plant growth promotion.

### Secondary metabolite gene clusters

FungiSMASH analysis identified 18 different types of secondary metabolites biosynthesis gene clusters in the genome of *T. trachyspermus* (Tables 3 and 4), including Type I polyketide synthases s, terpenes, nonribosomal peptide synthetases, nonribosomal peptide synthetases-like, β-lactones, fungal-RiPP-like, and hybrid biosynthesis gene clusters, including T1PKS/NRPS and T1PKS/NRPS-like. Forty-six biosynthesis gene clusters were detected in this studied genome (Table 3). Four BGCs were identified to share 100% similarity to known BGCs that encode for fusarin, dimethylcoprogen, YWA1, and choline biosynthesis. Four BGCs were identified to share at least 40% but less than 100% similarity to known BGCs that are deposited in the MIBiG repository, including squalstatin S1, nidulanin A, phomoidride, and phyllostictine A/phyllostictine B (https://mibig.secondarymetabolites.org). A further 10 BGCs were identified to share 7 to 33% similarity to known BGCs (Table 4). Most of the BGCs could not be identified in our studied species. The most abundant secondary metabolite biosynthetic gene cluster in the studied genome belonged to Type 1 polyketide synthases (T1PKSs).

**Table 3.** The number of times that each type of secondary metabolite biosynthetic gene cluster (SM-BGC) was detected in the genome of *Talaromyces trachyspermus* strain IRAN 3054C by FungiSMASH analysis.

	SM-BGC type	The number of times each BGC was detected.
1	NRPS-like	8
2	T1PKS	14
3	NRPS	6
4	T1PKS, NRPS	2
5	T1PKS, NRPS-like	2
6	Terpene	3
7	β-lactone	1
8	Other	1
9	Fungal—RiPP-like	9
	Total	46

**Table 4.** The putative secondary metabolites that were detected in the genome of *Talaromyces trachyspermus* by fungiiSMASH analysis.

BGC type	Length (nt)	Most similar compound	MIBiG accession	Similarity (%)
Terpen	21,484	Squalestatin S1	BGC0001839	60
NRP	49,455	Nidulanin A	BGC0001699	50
Polyketide	48,197	Phomoidride	BGC0001913	46
NRP + Polyketide	46,189	Fusarin	BGC0000064	100
NRP + Polyketide	50,496	Phyllostictine A/phyllostictine B	BGC0001741	40
NRP + Polyketide	52,406	Phyllostictine A/phyllostictine B	BGC0001741	30
Polyketide	45,991	Ywa1	BGC0002175	100
NRP	26,233	Dimethylcoprogen	BGC0001249	100
NRP	43,831	Choline	BGC0002276	100
Polyketide	43,912	Chromane	BGC0001907	33
Polyketide	47,709	Depudecin	BGC0000046	33
NRP	41,667	Metachelin	BGC0002710	25
NRP + Alkaloid + Polyketide:Iterative type I polyketide	51,435	Ucs1025a	BGC0001449	23

The similarity percentages of lower than 20% have not been included in this table.

## Discussion

Comparing quality metrics such as the number of contigs, N50, and N75 between the assembled genome in this study and the reference genome of *T. trachyspermus* strain 4041 reveals differences in size and quality. The assembly size obtained is 31.3 Mb, smaller than the reference genome size of 32 Mb. The reference genome was sequenced with Oxford nanopore technology, and the genome coverage was 57.0×, whereas Illumina platform with coverage of 20× was used in this study. The reference genome consists of 14 contigs and does not include assembled chromosomes with N50 of 3.8 Mb. The same determinants for our assembly were 552 contigs and N50 of 0.386 Mb, respectively. The completeness difference between the 2 genome assemblies may be attributed to variations in sequencing platforms, sequencing depth, and assembly techniques. Compared to long-read techniques such as Oxford Nanopore or PacBio, “short-read-based” technologies such as Illumina produce genome assemblies with smaller values of N50 and higher counts of contigs (Rayamajhi et al. 2022). Also, low depth or coverage of reads may result in some gaps in the assembly; consequently, some parts of the genome may not be represented in the genomic data (Baker 2012). This problem occurs particularly in genomic regions with high GC content and regions with high repeats (Ross et al. 2013).

Among the predicted secondary metabolite biosynthetic gene clusters identified, polyketide synthases (T1PKS) and Nonribosomal peptide synthetases (NRPS/NRPS-like) had the highest frequency. Polyketides have a large variety and several polyketides have been reported to be produced by *Talaromyces* species. Among them, a new derivative of spiculisporic acid, spiculisporic acid E, was isolated from the culture of *T. trachyspermus* strain KUFA 0021 (Kumla et al. 2014). Polyketides production has been reported by other *Talaromyces* species, including *T. lutcus*, *T. luteus*, *Talaromyces* sp., *T. wortmanii, T.ardifaciens, T. helices*, and *T. flavus (*Zhai et al. 2016).

The production of 2 cyclic peptides, talaromins A and B, has been reported from the endophytic fungus *T. wortmannii* (Zhai et al. 2016). In our genome analysis, fungiSMASH predicted several putative secondary metabolite gene clusters with varying degrees of similarity (7 to 100%) to recognized metabolites in the MIBiG database. A similar FungiSmash analysis was conducted for the genome assembly of *Talaromyces* sp. DC2 revealed the existence of Choline, YWA1, and Squalestatin S1 in the genome of that isolate which is consistent with our results. They identified 20 secondary metabolite biosynthetic gene clusters (Quan et al. 2024). Genome assembly of *T. pinophilus* strain 1 to 95 revealed 68 secondary metabolism gene clusters, mainly belonging to T1 polyketide synthase genes and nonribosomal peptide synthase genes (Li et al. 2017). In another research, 62 gene clusters (724 genes) involved in the secondary metabolism, including 18 Type I polyketide synthases (type 1 PKS), 15 nonribosomal peptide synthetase-like, nine nonribosomal peptide synthetases, 9 terpenes, 4 NRPS-T1PKS, 4 NRPS-like-T1PKS, 1 *β*-lactone, 1 NRPS-*β*-lactone, and one other, were identified in the genome of *T*. *albobiverticillius*. Among their identified secondary metabolites, 6 PKS genes were found with 100% similarity in the Type I polyketide synthases gene cluster. YWA1 was in common with our study (Wang et al. 2023). Genome analysis of *T. verruculosus* SJ9 showed that this genome contained 19 clusters in Type I polyketide synthase, 3 clusters encoding non-ribosomal peptide synthase cluster, 8 clusters in terpene, and 13 clusters in NRPS-like (Fu et al. 2024). The nidulanin A gene cluster is conserved in *Aspergillus* and *Penicillium* species (Gonçalves et al. 2021). This compound has yet to be tested for antimicrobial or virulence-related properties (Raffa and Keller 2019). Fusarins are mycotoxins produced by the genus Fusarium. Among the predicted putative metabolites in our research, there were some clusters that had good similarity with known biosynthesis gene clusters. The fusarin cluster was predicted with 100% similarity, even though fusarins have not yet been reported in Talaromyces or Penicillium species. This finding should be subjected to further investigation to see if these genes are indeed functional in *T. trachyspermus*. Similarly, YWA1 (an antiviral, antimicrobial, and insecticidal naphtho-γ-pyrone compound) and dimethylcoprogen (an antibacterial and antifungal siderophore compound) were both predicted at 100% similarity.

One compound belonging to the putative terpenes was identified in the *T. trachyspermus* draft genome showing similarity to known compound: qualestatin S1 (SQS1). SQS1, or zaragozic acid (ZA), is a fungal metabolite with broad antifungal activity. It is also a lead compound for cholesterol-lowering drugs (Lebe 2020).

Lower-similarity clusters were also discovered for phyllostictine A/B (30 to 40%). While phyllostictines have been studied as herbicides (Evidente et al. 2008; Zonno et al. 2008), their predicted presence in *T. trachyspermus* is based solely on sequence similarity and remains highly speculative. The low similarity rates in the majority of cases suggest these clusters may reflect encoding of alternative or novel metabolites, or may reflect draft genome assembly limitations. It is important to note that all the metabolites presented here are putative predictions derived through genome analysis. Metabolomic profiling or chemical validation has not yet been performed for this isolate. Furthermore, previous pathogenicity and phytotoxicity assays with this isolate on tomato and other hosts were not phytotoxic (Hemmati and Gholizadeh 2019; unpublished data), confirming its safety as a biocontrol agent. However, because the fungiSMASH similarity scores vary significantly (7 to 100%), experimental metabolomic investigations are needed to determine whether *T. trachyspermus* actually produces such metabolites, and, if so, to elucidate their biological activities and ecological functions.

In comparison to mycoparasitic fungus *T. rugulosus* (Wang et al. 2020) and mycoparasitic *T. pinophilus* (Li et al. 2017), respectively with 107 and 81 copies in their genome, the studied isolate's genome contains a higher number of fungal “cell wall degrading enzymes”, including chitinases, glucanases, peptidases, proteases, and lipases, which are essential for antagonistic activity. Additionally, the number of “chitinase encoding genes” in the genome of our studied isolate is higher than those detected in 3 commercial biocontrol species of Trichoderma, *T. harzianum*, *T. atroviride*, and *T. reesei* (Rosolen et al. 2023). This isolate also contains a high number of phosphatase coding sequences and 5 domains for siderophore synthase proteins in its genome. Phosphate insolubilization and siderophore production are significant mechanisms of plant growth promotion. Previous studies have reported the production of these enzymes and phosphate insolubilization by *T. trachyspermus* (Sahu et al. 2019). Plant growth promotion activity has also been reported by another *Talaromyces* biocontrol species, *T. pinophilus*, which has promoted rice growth (Khalmuratova et al. 2015). Genome analysis of several other species of *Talaromyces* has also revealed that *Talaromyces* species have gene clusters associated with “cell wall degrading enzymes.” Whole-genome sequencing of *T. piceus* strain 9-3 showed that its genome had different lignocellulolytic enzymes, including 2 cellobiohydrolases, 10 β-glucosidase, and 1 endo-β-1,4-glucanase gene cluster (He et al. 2017). *T. pinophilus* strain 1 to 95 contained a high number of CAZYmes in its genome, including 8 β-1,4-endoglucanases, 2 cellobiohydrolases, 29 β-glucosidases, 97 hemicellulose-degrading enzymes, and 24 α-amylases (Li et al. 2017). The high number of glucosidase and glucanases in the genome of other *Talaromyces* species is consistent with our findings. *Talaromyces* sp. strain DC2 contained 653 CAZymes responsible for plant cell wall degradation; among them, β-glucosidases and β-galactosidases had 27 genes and 23 genes in the genome. The most abundant glycoside hydrolases (GH) belonged to chitinases (Quan et al. 2024). Study on genome assembly of *T*. *albobiverticillius* revealed that glycosed hydrolases, including chitinases (GH18), *β*-glucosidases (GH3), and polygalacturonases (GH28), were the most frequent CAZYmes in its genome (Wang et al. 2023). We also report high copies of chitinases in our studied genome. Studies on CAZYmes of most Talaromyces species have mainly focused on plant cell wall degradation, as they have studied the isolates for plant debris degradation potential, whereas, in current research, we searched our studied genome for enzymes effective on fungal cell wall hydrolyzing, plant cell wall degradation, and mycoparasitism. A chromosome-level genome assembly of *Talaromyces rugulosus* was generated using a combination of PacBio long-read and Illumina paired-end data. *T. rugulosus* is a powerful enzyme producer and also a promising biocontrol agent against *Aspergillus flavus,* a notorious mycotoxin-producing plant pathogen (Wang et al. 2020). Their results showed that the genome of *T. rugulosus* is rich in genes encoding proteases, carbohydrate-active enzymes, fungal “cell wall degrading enzymes,” and secondary metabolite biosynthetic genes, demonstrating its mycoparasitic capability (Wang et al. 2020).

## Conclusion

Although the number of secondary metabolite biosynthesis gene clusters in our studied draft genome is lower in comparison to *T. pinophilus* (Li et al. 2017), *T*. *albobiverticillius* (Wang et al. 2023), and *T. rugulosus* (Wang et al. 2020) with the numbers of 68, 62, and 67 secondary metabolite biosynthesis gene clusters, respectively, the genome of *T. trachyspermus,* isolate IRAN 3054C, is rich in secondary metabolite gene clusters, compared to some other previous works on the genus Talaromyces, including *Talaromyces* sp. (Quan et al. 2024) and *T. verruculosus* (Fu et al. 2024), respectively with 20 and 43 identified SM-BGCs. This draft genome also has encoding genes for proteases and fungal “cell wall degrading enzymes,” reflecting its mycoparasitic potential against plant pathogenic fungi. Its genome contains sequences similar to genes encoding antifungal and herbicidal secondary metabolites, which is consistent with the in vitro and greenhouse observations reported for this isolate against *O.ramosa* (Hemmati and Gholizadeh 2019 and unpublished data). YWA1 and dimethylcoprogen, as siderophores with antifungal and antibacterial activity, were detected in the genome with 100% similarity. Other antifungal or antimicrobial secondary metabolites with a similarity of <100% are squalestatin S1 (60%) and nidulanin A (50%). Therefore, for the last groups of secondary metabolites, it is necessary to conduct a metabolomics study to find out the exact components produced with this isolate. Phyllostictins (30 and 40%) are herbicidal secondary metabolites. According to the low similarity percentage, their production also needs to be investigated in a study of the metabolites of this isolate. Genome analysis showed that strain IRAN 3054C serves as a potential source of essential enzymes for antagonistic activity against fungal pathogens. Although its genome harbors other CAZYmes for plant cell wall degradation, genes encoding mycoparasitism-related features are particularly prevalent. However, the mechanism underlying its biocontrol activity against broomrape (*O.ramosa*) remains unknown, and further investigations are required to clarify its specific herbicidal mechanisms.

## Data Availability

Sequence data that support the findings of this study have been deposited in DDBJ/ENA/GenBank under the accession JBNAEF000000000. The Fasta and Gff3 files can be found at Figshare (https://doi.org/10.6084/m9.figshare.30651500).
